# Anti-neuraminidase antibodies against pandemic A/H1N1 influenza viruses in healthy and influenza-infected individuals

**DOI:** 10.1371/journal.pone.0196771

**Published:** 2018-05-09

**Authors:** Yulia Desheva, Ivan Sychev, Tatiana Smolonogina, Andrey Rekstin, Natalia Ilyushina, Vladimir Lugovtsev, Anastasia Samsonova, Aleksey Go, Anna Lerner

**Affiliations:** 1 Virology Department, Federal State Budgetary Scientific Institution “Institute of Experimental Medicine”, Saint Petersburg, Russian Federation; 2 Department of Fundamental Problems of Medicine and Medical Technologies, Saint Petersburg State University, Saint Petersburg, Russian Federation; 3 Division of Biotechnology Research and Review II, Center for Drug Evaluation and Research, U.S. Food and Drug Administration, Silver Spring, Maryland, United States of America; 4 Division of Viral Products, Center for Biologics Evaluation and Research, Food and Drug Administration, Silver Spring, Maryland, United States of America; 5 Clinical and Diagnostic Center, Research Institute of Influenza, Saint Petersburg, Russian Federation; 6 Medical Research Center, Federal State Budgetary Scientific Institution “Institute of Experimental Medicine”, Saint Petersburg, Russian Federation; Icahn School of Medicine at Mount Sinai, UNITED STATES

## Abstract

The main objective of the study was to evaluate neuraminidase inhibiting (NI) antibodies against A/H1N1pdm09 influenza viruses in the community as a whole and after infection. We evaluated NI serum antibodies against A/California/07/09(H1N1)pdm and A/South Africa/3626/2013(H1N1)pdm in 134 blood donors of different ages using enzyme-linked lectin assay and in 15 paired sera from convalescents with laboratory confirmed influenza. The neuraminidase (NA) proteins of both A/H1N1pdm09 viruses had minimal genetic divergence, but demonstrated different enzymatic and antigenic properties. 5.2% of individuals had NI antibody titers ≥1:20 against A/South Africa/3626/2013(H1N1)pdm compared to 53% of those who were positive to A/California/07/2009(H1N1)pdm NA. 2% of individuals had detectable NI titers against A/South Africa/3626/13(H1N1)pdm and 47.3% were positive to A/California/07/2009(H1N1)pdm NA among participants negative to hemagglutinin (HA) of A/H1N1pdm09 but positive to seasonal A/H1N1. The lowest NI antibody levels to both A/H1N1pdm09 viruses were detected in individuals born between 1956 and 1968. Our data suggest that NI antibodies against A/South Africa/3626/13 (H1N1)pdm found in the blood donors could have resulted from direct infection with a new antigenic A/H1N1pdm09 variant rather than from cross-reaction as a result of contact with previously circulating seasonal A/H1N1 variants. The immune responses against HA and NA were formed simultaneously right after natural infection with A/H1N1pdm09. NI antibodies correlated with virus-neutralizing antibodies when acquired shortly after influenza infection. A group of middle-aged patients with the lowest level of anti-NA antibodies against A/California/07/2009 (H1N1)pdm was identified, indicating the highest-priority vaccination against A/H1N1pdm09 viruses.

## Introduction

According to WHO-recognized National Influenza Centers in Moscow and Saint Petersburg, 2015/2016 epidemic season in Russia characterized by rapid increase of influenza and acute respiratory infection (ARI) morbidity from week 3 of 2016. Increases in influenza-like illness and hospitalization rates were similar to those observed during the 2009 pandemic and epidemic in 2010–2011 and were much higher than in other seasons [1]. According to the phylogenetic analysis of amino acid sequences of the main antigenic glycoprotein hemagglutinin (HA), many of A/H1N1 viruses isolated in Russia in 2015–2016 belong to the clade 6B of A/H1N1pdm09 (A/South Africa/3626/2013-like) [1]. Antibodies directed against HA provide the main protection against influenza illness. However, as it was shown in 1973, antibodies against minor immunogenic viral glycoprotein, neuraminidase (NA), can also provide protection against influenza infection [2]. Namely, antibodies against NA block viral progeny release from cells during detachment of mature viral particles from the cell surface. At the initial stage of influenza infection cycle, anti-NA antibodies may prevent connection of HA with cellular receptors [3], block proapoptotic NA function [4], and inhibit NA plasminogen activation [5]. Furthermore, antibodies against NA can facilitate recognition of infected cells by macrophages and natural killer (NK) cells, mediating the activation of the complement system during complement-dependent cytotoxicity [6].

The hemagglutination-inhibition (HI) assay is most commonly used to assess previous exposure and protective immunity to influenza viruses as well as to evaluate influenza vaccine immunogenicity. However, the presence of NA inhibiting (NI) antibodies is the least explored. The 2009 influenza pandemic caused by A/H1N1pdm09 initiated a number of studies on the antibodies against viruses containing the N1 NA. The importance of NA immunity against naturally occurring influenza was previously demonstrated by evaluating HI and NI antibody titers in a study conducted during 2009–2011 [7]. The presence of a population possessing the cross-reactive NI antibodies, which were acquired as a result of previous infection or vaccination with previously circulated influenza viruses, can be decisive in reducing illness and mortality, as shown during a pandemic caused by the A/H3N2 virus in 1968 [2]. In addition, a number of studies were conducted to identify cross-reactive anti-NA antibodies against the new influenza virus like A/H1N1pdm09 [8] and to examine the formation and function of protective antibodies against NA by immunization with influenza vaccines [9, 10]. At the WHO meetings in 2005 and 2009, leading experts noted the increasing importance of developing and improving methods for detection of antibodies against influenza virus NA [11]. It is important to study immunity against NA because NI antibodies are considered to be independent predictors of anti-influenza immunity [7, 8]. The aim of the present study was to evaluate serum anti-NA antibodies against two A/H1N1pdm09 strains, A/California/07/09 (H1N1)pdm and A/South Africa/3626/2013 (H1N1)pdm, in the Russian population.

## Materials and methods

### Viruses

To evaluate the NI antibodies against A/H1N1pdm09 viruses, we developed chimeric viruses with HA non-relevant to seasonal influenza viruses. The A/H7N1 reassortant virus containing HA from A/horse/Prague/1/56 (H7N7) and NA from A/California/07/2009 (H1N1)pdm was prepared using classical genetic reassortment in developing chick embryos as described elsewhere [12]. The A/H6N1 virus harboring HA gene from A/herring gull/Sarma/51c/2006 (H6N1) and NA from A/South Africa/3626/13 (H1N1)pdm was generated using a standard plasmid-based genetic technique [13].

Influenza A/horse/Prague/1/56 (H7N7) virus and the live-attenuated A/H1N1 reassortant with a 6:2 genome composition carrying HA and NA genes from A/South Africa/3626/13 (H1N1)pdm [14] were provided by the Centers for Disease Control and Prevention (CDC) (Atlanta, USA). The wild-type A/South Africa/3626/2013 (H1N1)pdm virus was obtained from the National Institute for Biological Standards and Control (NIBSC, UK) repository. The A/herring gull/Sarma/51c/2006 (H6N1) virus was supplied by the Institute of Influenza, Saint Petersburg, Russia. A/California/07/2009 (H1N1)pdm, A/New Caledonia/20/99 (H1N1) (A/NC) and A/Puerto Rico/8/34 (H1N1) (A/PR/8) were provided by the Institute of Experimental Medicine. All viruses were grown in the allantois cavity of 10-day developing chicken embryos supplied by “Sinyavino” poultry farm (Kirovsk Area, Leningrad Region, Russia) at the 33°C for 48 h, aliquoted, and stored at -70°C.

### Sequence analysis

The DNA-copies of the viral RNA segments were obtained using the OneStep RT-PCR Kit (Qiagen, Netherlands). Following the electrophoresis of the DNA-copies in 1.5% agarose gel and consecutive purification by QIAquick PCR purification Kit (Qiagen), the sequencing was conducted on DNA-analyzer ABI 3730xl using the BigDye Terminator v3.1 Cycle Sequencing kit (Applied Biosystems, USA). The processing of the nucleotide sequence data was performed using the 3730 Data Collection v3.0 software package (Applied Biosystems).

### Molecular analysis

Multiple alignments and the identity analysis of the NA amino acid sequences from various A/H1N1 influenza viruses were carried out using the GeneDoc program, version 2.6.002. The amino acid sequences of the NA proteins were obtained from GenBank and the Global Initiative on Sharing All Influenza Data (GISAID) database. Nucleotide sequences used in this study are available under the following accession numbers: FJ984386, KF648099, KU509643.1, AF250356, CY033624, AB671290, CY022071, EU124136, CY058489, CY044367, CY022351, AF250363, CY061831, GU984385, GU367316, GQ183619, CY050881, HM189306, GU984412, FJ798780, EU045389, GQ484356. Computer analysis was carried out using UGENE software from Unipro (Russia, Novosibirsk) [15].

### NA enzyme activity and kinetics

The NA activity of influenza H1N1 viruses was measured by a fluorescence-based assay using the fluorogenic substrate MUNANA (Sigma-Aldrich, USA), based on the method of Potier et al. [16] as described previously [17]. Briefly, A/California/07/09 (H1N1)pdm and A/South Africa/3626/2013 (H1N1)pdm viruses were standardized to an equivalent NA protein content of 0.015 ng/μl as determined by protein gel electrophoresis using purified and concentrated viruses. This virus dilution was selected as a dilution that converted ≤15% MUNANA substrate to product during the reaction time in order to meet the requirements for steady-state kinetic analysis [17]. Virus dilutions were prepared in enzyme buffer [32.5 mM of 2-(N*-*morpholino) ethanesulfonic acid (MES), 4 mM of calcium chloride, pH 6.5] and added (100 μl/well) in duplicate to a flat-bottom 96-well opaque black plate (Corning, USA). After preincubation for 20–30 min at 37°C, the MUNANA substrate at various concentrations (separately pre-incubated for 20–30 min at 37°C) was added to all wells (50 μl/well). Immediately after adding the MUNANA substrate, the plate was transferred to a 37°C pre-warmed SpectraMAX Gemini XPS microplate reader (Molecular Devices, USA) and fluorescence was measured every 60 s for 60 min at 37°C, using excitation and emission wavelengths of 360 nm and 460 nm, respectively. Enzymatic reactions were performed under conditions where signal-to-noise ratios were above 10 during more than 30 min of the reaction time. Time course data from each concentration of the MUNANA substrate were examined for linearity by linear regression analysis. Data with *R*^2^>0.99 were used for analysis. The kinetic parameters Michaelis-Menten constant (*K*_m_) and maximum velocity of substrate conversion (*V*_max_) of the NAs were calculated by fitting the data to the appropriate Michaelis-Menten equations by using nonlinear regression in Prism 6.0 software (GraphPad Software, USA). Values are the means of three independent determinations.

### Ethics statement

The study involved a retrospective analysis of participant serum samples left from routine tests. Blood serum samples were collected from the 134 patients of the Institute for Experimental Medicine Medical Research Center for routine tests from January 6, 2016 till April 1 2016. These serum samples included 16 sera from 24–39 years old persons, 31 sera from 40–59 years old participants and 87 sera from patients aged 60–84 years. None of participants was vaccinated against influenza in 2016. Left sera were stored at -20°C. After receiving the approval from the Ethics Committee of the Institute for Experimental Medicine No. 2/16 of May 12, 2016, these sera were provided to study neuraminidase antibodies. We also tested the 42 archive serum samples obtained from non-vaccinated volunteers 20–59 years old in October 2010. The 15 paired sera from the convalescents with laboratory confirmed A/H1N1pdm09 influenza infections collected in January-February 2016 at hospitalization and 4–8 days later were provided by The Institute of Influenza. The age of these patients was 19 to 83 years. Written informed consent was obtained for each participant. The participants were fully informed of the research procedures and any risks associated with participation, and consented to participate in scientific projects.

None of the authors collected the samples used in the study, had access to information that would allow the identification of individual patients during the extraction of data from medical records, the data was de-identified before access by any of the authors.

### Detection of serum antibodies against A/H1N1 viruses

HI test with blood sera was performed using a 0.75% suspension of human red blood cells (Group “0”) in “U”-bottom 96-well polymer plates for immunological reactions using standard procedures [18]. To remove thermo-labile inhibitors, the sera were heated at 56°C for 30 min. To remove thermo-stable hemagglutination inhibitors, the studied sera were treated with an extract of *Vibrio cholerae* NA (Denka Seiken Co., Japan), and tested in duplicate for HI antibodies with the following test antigens: A/South Africa/3626/13 (H1N1)pdm, A/California/07/2009 (H1N1)pdm, A/New Caledonia/20/99 (H1N1), and A/Puerto Rico/8/34 (H1N1). The titers of HI antibodies were expressed as the reciprocal of the highest serum dilution at which HI was observed.

The production of anti-NA antibodies against A/California/07/2009 (H1N1)pdm and A/South Africa/3626/13 (H1N1)pdm was evaluated in the sialidase activity inhibition test [19] using the A/H7N1 and A/H6N1 reassortant viruses described above after purification and concentration on a stepwise 30/60% sucrose gradient. To assay anti-NI antibodies, 96-well plates (Sarstedt AG & Co, Germany) were coated overnight with 150 μL of 50 μg/mL fetuin (Sigma-Aldrich, USA). The purified A/H7N1 or A/H6N1 reassortants were adjusted in phosphate-buffered saline (PBS) containing 1% bovine serum albumin (BSA) to obtain 128 hemagglutination units (HAU) and yielded 0.4–0.6 optical density at 450 nm (OD_450_). 65 μL of sera samples were heated at 56°C for 30 min, serially diluted in PBS-BSA, and incubated with an equal volume of pre-diluted virus for 30 min at 37°C. After incubation, 100 μL of the mixture was added to the fetuin-coated wells. After 1 h incubation at 37°C, the plates were washed, and NA activity was measured by incubating with peroxidase-labeled peanut lectin (2.5 μg/mL, Sigma-Aldrich, USA) for 1 h at room temperature followed by washing and adding 100 μL of peroxidase substrate. The reaction was stopped after 5 min with 100 μL of 1 N sulfuric acid. OD values were measured at 450 nm using the universal microplate reader (ELx800, Bio-Tek Instruments Inc, USA). The titer of serum NI antibodies was calculated as the reciprocal dilution of the sample with 50% inhibition of NA activity, *i*.*e*. two-fold decrease in optical density in comparison with the virus control wells.

Determination of neutralizing antibodies against A/South Africa/3626/13 (H1N1)pdm was carried out using the microneutralization (MN) test in the Madin-Darby canine kidney (MDCK) cells with no-RDE-treated sera as described previously [18].

### Statistical analysis

Data were analyzed using Statistica software, version 6.0 (StatSoft Inc., USA). The antibody titers were expressed as log2 of the inversed final dilution for statistical analysis. Geometric mean titers (GMT), medians (Me) and lower and upper quartiles (Q1; Q3) were calculated and used to represent the antibody levels. Comparisons of two independent groups were made with nonparametric Kolmogorov-Smirnov 2-sample test. To compare multiple independent groups, we used a Kruskal-Wallis analysis of variance (ANOVA) with subsequent multiple pairwise comparisons based on Kruskal-Wallis sums of ranks. Comparisons of two dependent variables were performed using Wilcoxon matched pairs test. Fisher exact 2-tailed test was performed in case of nominal variables. Non-parametric measure of statistical dependence between 2 variables was done using Spearman’s rank correlation coefficient (r). The *P*-value < 0.05 was considered to be statistically significant.

## Results

### Analysis of NA amino acid sequences of A/H1N1 viruses

**Fig 1:**
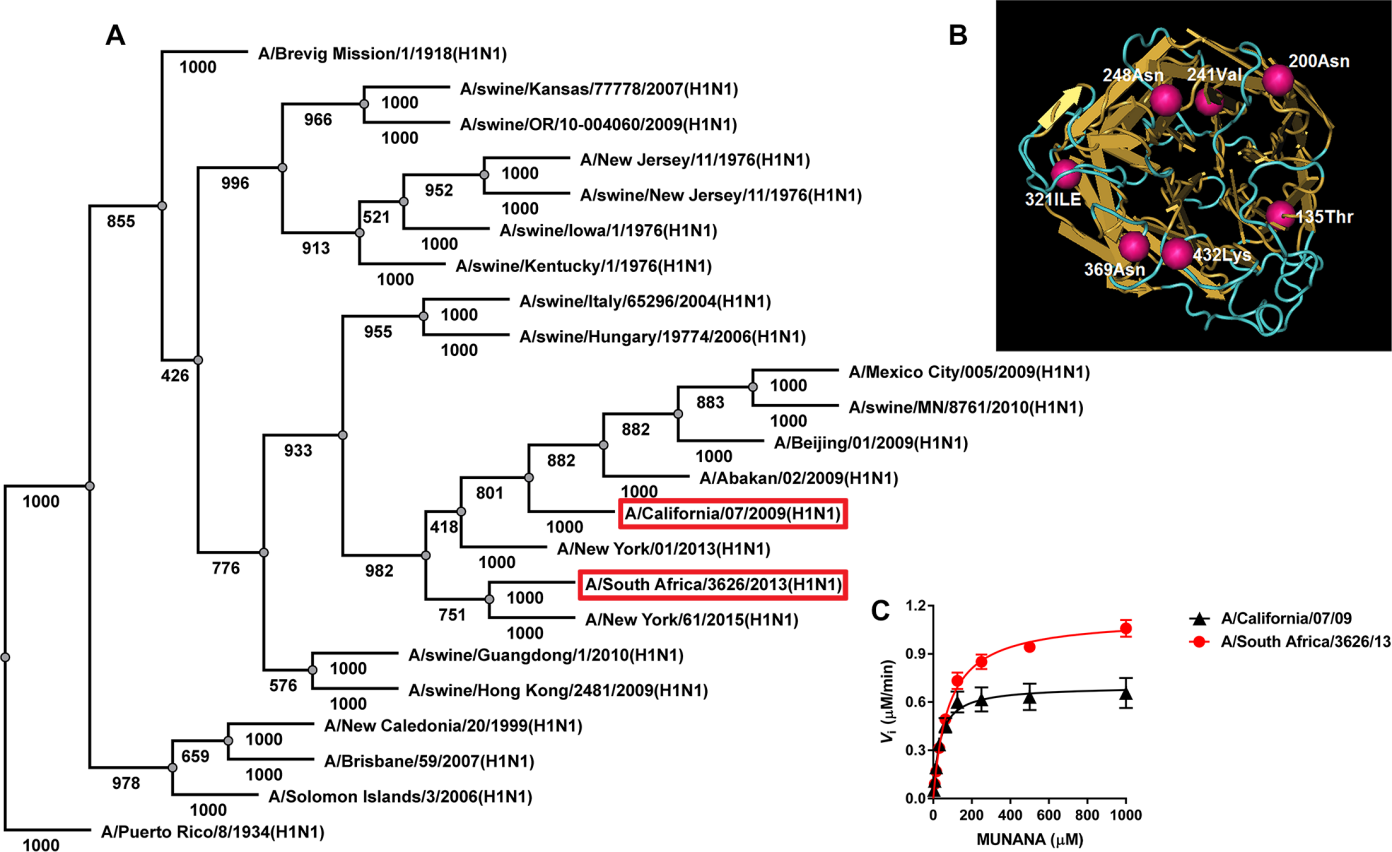
Analysis of N1 NA proteins. (A) Phylogenetic analysis of NAs originating from different A/H1N1 viruses isolated from human and swine (AA 1–469). Phylogenetic analysis of amino acid sequences of NAs originating from different A/H1N1 viruses isolated from human and swine (AA 1–469). The sequences were obtained from The NCBI Influenza Virus Sequence Database. Numbers of bootstrapping trees next to each node represent a measure of support for the node. (B) Three-dimensional model of the NA “head” (AA 83–469) was created using Cn3D software. Seven amino acid substitutions that differ in the NA head domain of A/California/07/09 (H1N1)pdm compared to A/South Africa/3626/13 (H1N1)pdm are shown in purple. (C) NA enzyme kinetics of A/California/07/09 (H1N1)pdm and A/South Africa/3626/13 (H1N1)pdm viruses. Substrate conversion velocity (*V*_i_) of NA was measured as a function of substrate concentration.

We used a distance method of proximity for the reconstruction of NA phylogenetic trees. Based on the initial alignment we evaluated the stability of the tree topology using bootstrap analysis using the results of the construction and comparison of phylogenetic trees generated for 1,000 sets of NA amino acid sequences. Our phylogenetic analysis of N1 amino acid sequences demonstrated that the NA of A/California/07/09 (H1N1)pdm as well as the NA of other viruses isolated after 2009 in North America and Asia exhibited similarity to the phylogenetic branch of the swine viruses of the Euro-Asian lineage isolated in 2004 (Fig 1A). NA sequences of recent epidemic A/H1N1 viruses were more closely related to A/H1N1 strains isolated in 1918 and 1943 compared with the A/H1N1pdm09 isolates. The NAs of A/California/07/09 (H1N1)pdm and A/South Africa/3626/13 (H1N1)pdm were 98% identical and differed only by 10 amino acid substitutions, I34V, L40I, N44S, T135A, N200S, V241I, N248D, I321V, N369K, K432E (N1 numbering here and throughout the text), which do not alter NA polarity and/or charge. Mutations at residues 34, 40, and 44 were located in the NA stem, whereas the rest were located in the head domain outside the active center (Fig 1B) [20].

We next determined NA activities of A/California/07/09 (H1N1)pdm and A/South Africa/3626/13 (H1N1)pdm viruses by measuring NA enzyme *K*_*m*_ *and V*_max_ values for both A/H1N1pdm09 strains and by using the fluorogenic MUNANA as a substrate (Fig 1C and Table 1). Our data showed that NA protein of A/South Africa/3626/13 (H1N1)pdm exhibited significantly higher affinity for the substrate (mean *K*_m_, 2.3-fold) than A/California/07/09 (H1N1)pdm NA (*P* < 0.05). Furthermore, NA enzyme activity of A/South Africa/3626/13 (H1N1)pdm was significantly higher compared to that of A/California/07/09 (H1N1)pdm (*V*_max_ ratio = 1.6; Fig 1C and Table 1).

**Table 1:** Enzymatic properties of A/H1N1pdm09 NAs.

Viruses	*V*_max_ (μM/min)^a^	*K*_m_ (μM)^b^
A/California/07/09 (H1N1)pdm	0.70 ± 0.02	34.35 ± 3.77
A/South Africa/3626/13 (H1N1)pdm	1.13 ± 0.02*	78.45 ± 5.49*

^*a*^ The *V*_max_ was calculated using a nonlinear regression of the curve according to the Michaelis-Menten equation.

^*b*^ The *K*_m_ represents the Michaelis-Menten constant (μM) at which the reaction rate is half of *V*_max_. The enzyme kinetic data were fit to the Michaelis-Menten equation using GraphPad Prism, version 6.0. Values are the means ± standard deviations from three independent determinations.

*P < 0.05, compared to the values for the A/California/07/09 (H1N1)pdm virus by *t*-test.

### Comparison of “herd” immunity against A/California/07/09 (H1N1)pdm in 2010–2011 and 2015–2016 epidemic seasons

**Fig 2:**
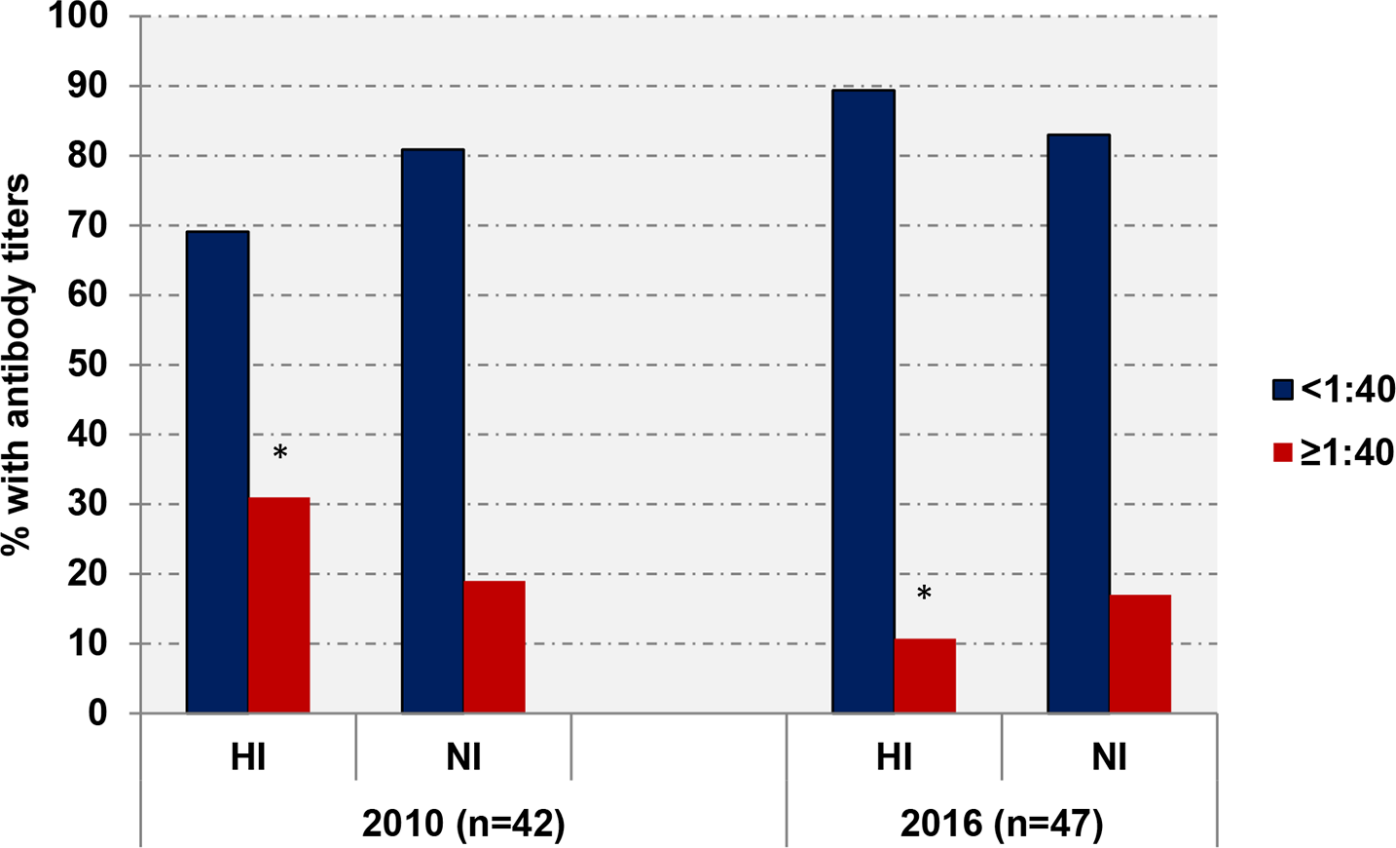
The levels of “herd” immunity against A/California/07/09 (H1N1)pdm virus in 2009–2010 and 2015–2016 epidemic seasons among participants 20–59 years old. *, *P* = 0.047.

We compared the HI and NI antibody levels against A/California/07/09 (H1N1)pdm in the sera collected from adult volunteers between 20–59 years of age. The sera were collected in October 2010 (n = 42) and in January-March 2016 (n = 47). ANOVA test showed no difference in age distribution of the participants between both groups (*P* > 0.05). As seen in Fig 2, the proportions of with NI antibody titers <1:40 and ≥1:40 were similar between volunteers examined in 2010 (*i*.*e*., one year after A/H1N1pdm09 emerged) and in 2016 (*i*.*e*., after 6 years of A/H1N1pdm09 circulation).

Surprisingly, a number of subjects with HI antibody titers ≥1:40 against A/California/07/09 (H1N1)pdm (*i*.*e*., level that is traditionally associated with at least a 50% reduction in the risk of disease due to influenza infection) was significantly lower among volunteers examined in 2016 compared to 2010 (*P* = 0.047). These results may suggest decreased circulation of A/H1N1pdm09 in 2016 compared to 2010.

### Detection of HI and NI antibodies against A/South Africa/3626/13 (H1N1)pdm and A/California/07/09 (H1N1)pdm in 134 blood donors examined during the 2015–2016 epidemic season

**Fig 3:**
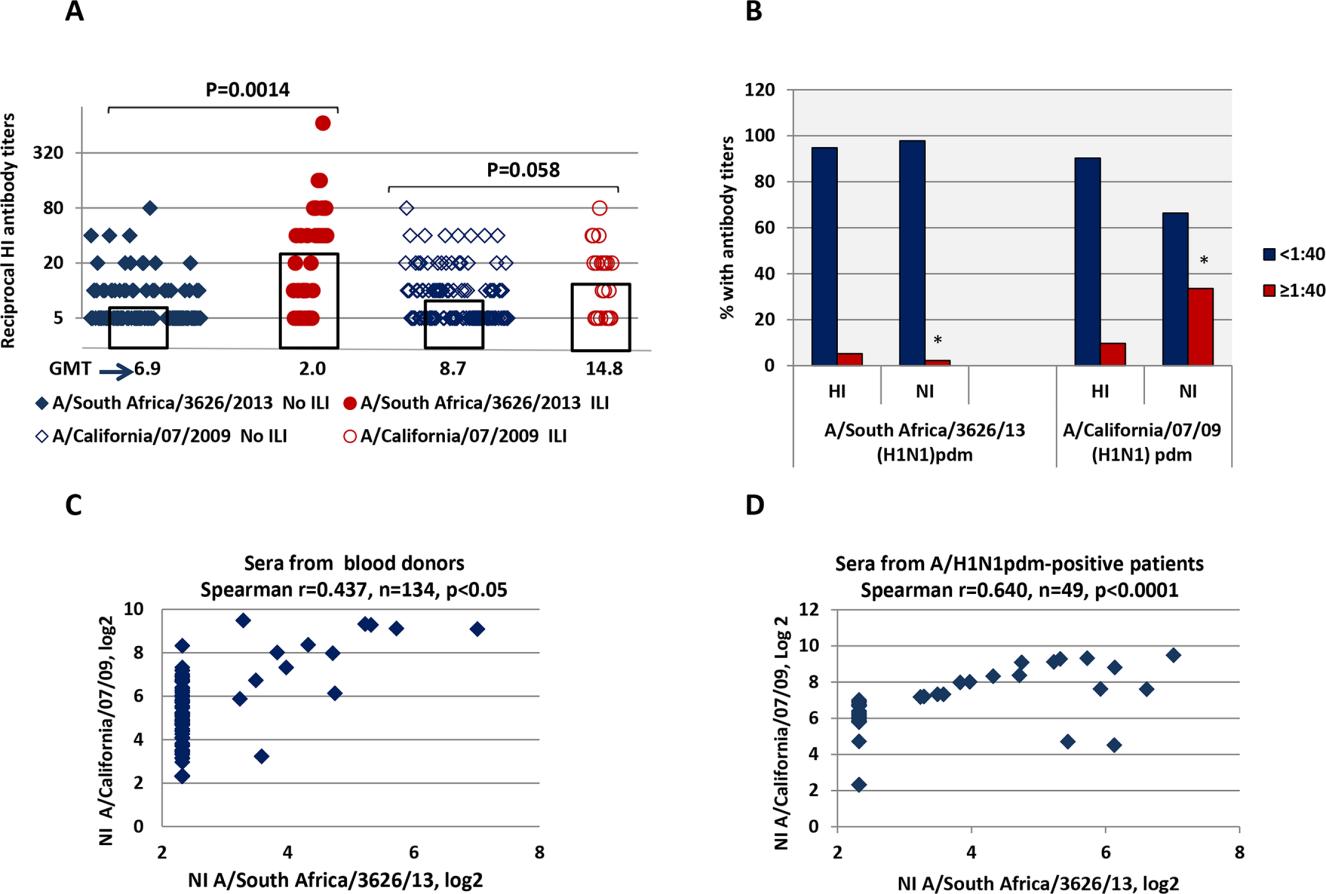
The antibody levels against A/H1N1pdm09 viruses in 134 blood donors 24–84 years of age examined in 2016. (A) HI antibody titers against A/H1N1pdm09 viruses among participants with registered ILI or without ILI. GMT were calculated and used to represent the mean antibody levels. (B) Proportion of subjects with HI and NI antibody titers against surface antigens originated from A/South Africa/3626/2013 (H1N1)pdm or A/California07/2009 (H1N1)pdm strains; *, proportion of subjects with NI antibody titers ≥ 1:40 against A/South Africa/3626/13 (H1N1)pdm and A/California/07/09 (H1N1)pdm differ significantly (*P* < 0.0001). (C) Correlation analysis of NI antibodies against A/California/07/09 (H1N1)pdm versus A/South Africa/3626/13 (H1N1)pdm among blood donors. (D) Correlation analysis of NI antibodies against A/California/07/09 (H1N1)pdm versus A/South Africa/3626/13 (H1N1)pdm among patients seropositive to A/H1N1pdm viruses.

The antibody levels against HA and NA of A/H1N1pdm09 viruses were examined among 134 patients of Medical Research Center. Since the examined subjects did not receive vaccination against A/H1N1pdm09, it was assumed that the antibodies to surface antigens of A/H1N1pdm09 viruses were acquired due to natural infection. Among all these subjects was obtained statistically significant differences between HI antibody levels to A/South Africa/3626/13 (H1N1)pdm in participants with influenza-like illnesses (ILI) compared with those with no ILI (*P* = 0.0014, Fig 3A). With respect to A/California/07/09 (H1N1)pdm these differences were not statistically significant (*P* = 0.058). These data may suggest that the drift A/H1N1pdm09 variant, presumably A/South Africa/3626/13-like, has already circulated in St. Petersburg along with A/California/07/09 (H1N1)pdm during the period of the study.

As seen in Fig 3B, among all 134 volunteers 24–84 years of age, the majority of participants had HI antibody titers <1:40 against A/South Africa/3626/13 (H1N1)pdm and A/California/07/09 (H1N1)pdm. We observed similar proportions of subjects possessing HI antibody titers ≥1:40 against A/South Africa/3626/13 (H1N1)pdm and A/California/07/09 (H1N1)pdm (5.2% and 9.7%; *P* > 0.05). Contrary data were obtained for NI antibodies: the NI antibody titers ≥1:40 against A/California/07/09 (H1N1)pdm were found in 33.6% of participants, whereas only 2.2% of subjects demonstrated such NI antibodies levels against A/South Africa/3626/13 (H1N1)pdm (*P* < 0.0001). The correlation between NI antibody titers against A/California/07/09 (H1N1)pdm and A/South Africa/3626/13 (H1N1)pdm among 134 blood donors was established as a link of medium strength: only 19.1% of the ranks variability in one variable can be explained using the ranks of another variable (r = 0.437, n = 134, *P* < 0.05, Fig 3C). The NI antibody titers against A/California/07/09 (H1N1)pdm and A/South Africa/3626/13 (H1N1)pdm better correlated (r = 0.640; n = 49; *P* < 0.0001) in the sera of the patients seropositive to A/H1N1pdm viruses (Fig 3D)

**Fig 4:**
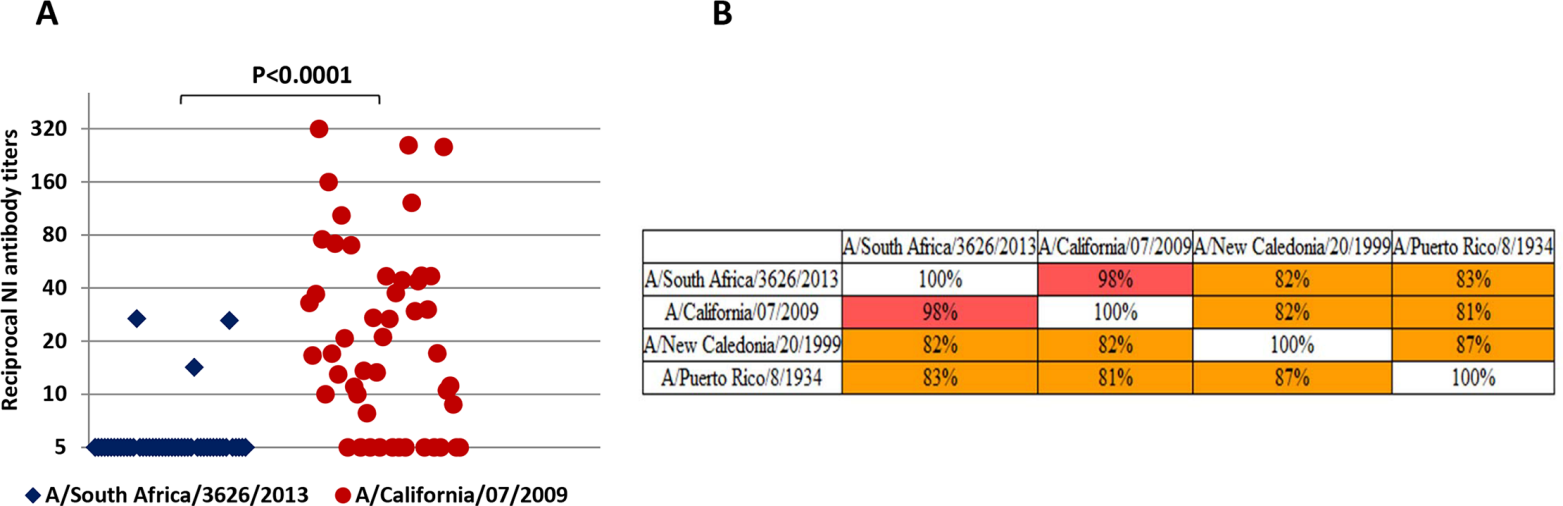
Analysis of the sera from 48 participants negative to HA of A/H1N1pdm09 viruses, but positive to A/New Caledonia/20/99 (H1N1) or A/Puerto Rico/8/34 (H1N1). (A) NI antibody levels against A(H1N1)pdm09 viruses. (B) Distance matrix of the A/H1N1 influenza viruses HA amino-acid sequences. Matrix shows identity between influenza virus strains.

The low NI antibody titers against A/South Africa/3626/13 (H1N1)pdm and high NI antibody titers against A/California/07/09 (H1N1)pdm may suggest that the drift variant A/South Africa/3626/13 (H1N1)pdm only started circulating in 2016. This finding may also indicate that antibodies against A/South Africa/3626/13 (H1N1)pdm NA found in the blood donors could result from direct infection with a new antigenic A/H1N1pdm09 variant rather than from cross-reaction as a result of contact with previously circulating seasonal A/H1N1 variants. Thus, antibody levels against A/South Africa/3626/13 (H1N1)pdm NA were significantly lower than against A/California/07/09 (H1N1)pdm NA among the 48 participants negative to HA of A/H1N1pdm09 viruses but positive to A/New Caledonia/20/99 (H1N1) or A/Puerto Rico/8/34 (H1N1) (*P* < 0.0001; Fig 4A) which HA amino-acid sequences differ more than 15% from A/California/07/09 (H1N1)pdm and A/South Africa/13 (Fig 4B). Since these patients did not possessed antibodies against HA of A/H1N1pdm09 viruses, it is likely that the detected antibodies to HA of previously circulated A/H1N1 viruses could be the result of antigenic ‘sin’. Despite the NA amino-acid sequences of A/South Africa/3626/13 (H1N1)pdm and A/California/07/09 (H1N1)pdm were very similar (Fig 1A), the levels of “herd” immunity against NA of A/H1N1pdm09 viruses in these patients varied significantly: NI antibody titers ≥1:40 were detected in 29.1% against A/California/07/09 (H1N1)pdm and were not found against A/South Africa/3626/13 (H1N1)pdm (P<0.01).

### Comparative analysis of the antibodies against A/H1N1 in patients of different ages

The age distribution of A/H1N1-specific antibodies was analyzed in several participant age groups: subjects born prior to 1957, when only A/H1N1 viruses were circulating; subjects born between 1957–1976 when A/H2N2 and A/H3N2 viruses were circulating, and subjects born in 1977 and later, when A/H1N1 re-emerged and began co-circulating along with A/H3N2 (Table 2).

**Table 2:** Antibodies against HA and NA of A/H1N1 viruses among 134 subjects of different ages examined in 2016.

Year of birth	No. of participants in the group	HI antibodies				NI antibodies	
		A/South Africa/ 3626/13 (H1N1)pdm	A/California/07/09 (H1N1)pdm	A/NC	A/PR/8	A/South Africa/ 3626/13 (H1N1)pdm	A/California/07/09 (H1N1)pdm
		(log2, Me, Q1; Q3)	(log2, Me, Q1; Q3)	(log2, Me, Q1; Q3)	(log2, Me, Q1; Q3)	(log2, Me, Q1; Q3)	(log2, Me, Q1; Q3)
1977 and later	16	2.8 (2.3; 3.8)	3.3 (2.3; 4.8)	4.3 (2.3; 5.3)*	2.8 (2.3; 3.3)	2.3 (2.3;2.3)	4.2 (4.2;5.6)
1957–1976	31	2.3 (2.3; 3.3)	3.3 (2.3; 4.3)	3.3 (2.3; 4.3)**	2.3 (2.3; 3.3)	2.3 (2.3; 3.3)	2.3 (2.3; 3.8)
Before 1957	87	2.3 (2.3; 3.3)	2.3 (2.3; 3.3)	2.3 (2.3; 3.3)	3.3 (2.3; 4.3)	2.3 (2.3; 2.3)	4.9 (3.4;6.4)***

Note: (Me–medians; Q1; Q3—lower and upper quartiles).

*, *P* = 0.023 compared to subjects born before 1957

**, *P* = 0.046 compared to subjects born before 1957

***, *P* = 0.00007 compared to subjects born in 1957–1976.

The highest levels of anti-HA antibodies against seasonal A/New Caledonia/20/99 (H1N1) and both A/H1N1pdm09 viruses were detected in the 24–39 age group compared to elder people (*P* = 0.023). Participants between 60–84 years of age demonstrated the lowest level of HI antibodies against A/New Caledonia/20/99 (H1N1), slightly increased HI titers against A/Puerto Rico/8/34 (H1N1) and the highest NI antibody titers against A/California/07/09 (H1N1)pdm compared with other groups (*P* < 0.0001) (Table 2). Our data confirm earlier findings that older people, who had been in contact with A/H1N1 viruses that have not been in circulation for a long time, may demonstrate antigenic “sin” when infected with recent viruses [21, 22]. In this case, the priming antibodies are formed not only to the infecting virus but also to the previous variants. However, the question of the protective role of these antibodies still remains unclear. On one hand, the presence of such antibodies can make a definite contribution to the protection against infection with a new antigenic variant [23]. On the other hand, there is evidence that pre-existing antibodies to previously circulating variants may somehow prevent the development of protective antibodies in infection with the new antigenic variants [24].

The lowest levels of NI antibodies against A/California/07/09 (H1N1)pdm and no NI antibodies against A/South Africa/3626/13 (H1N1)pdm were found in people 40–59 years old who were born from 1956 to 1976, when the H2N2 and H3N2 viruses were in circulation (Table 2).

### Analysis of sera samples from the convalescents

**Fig 5:**
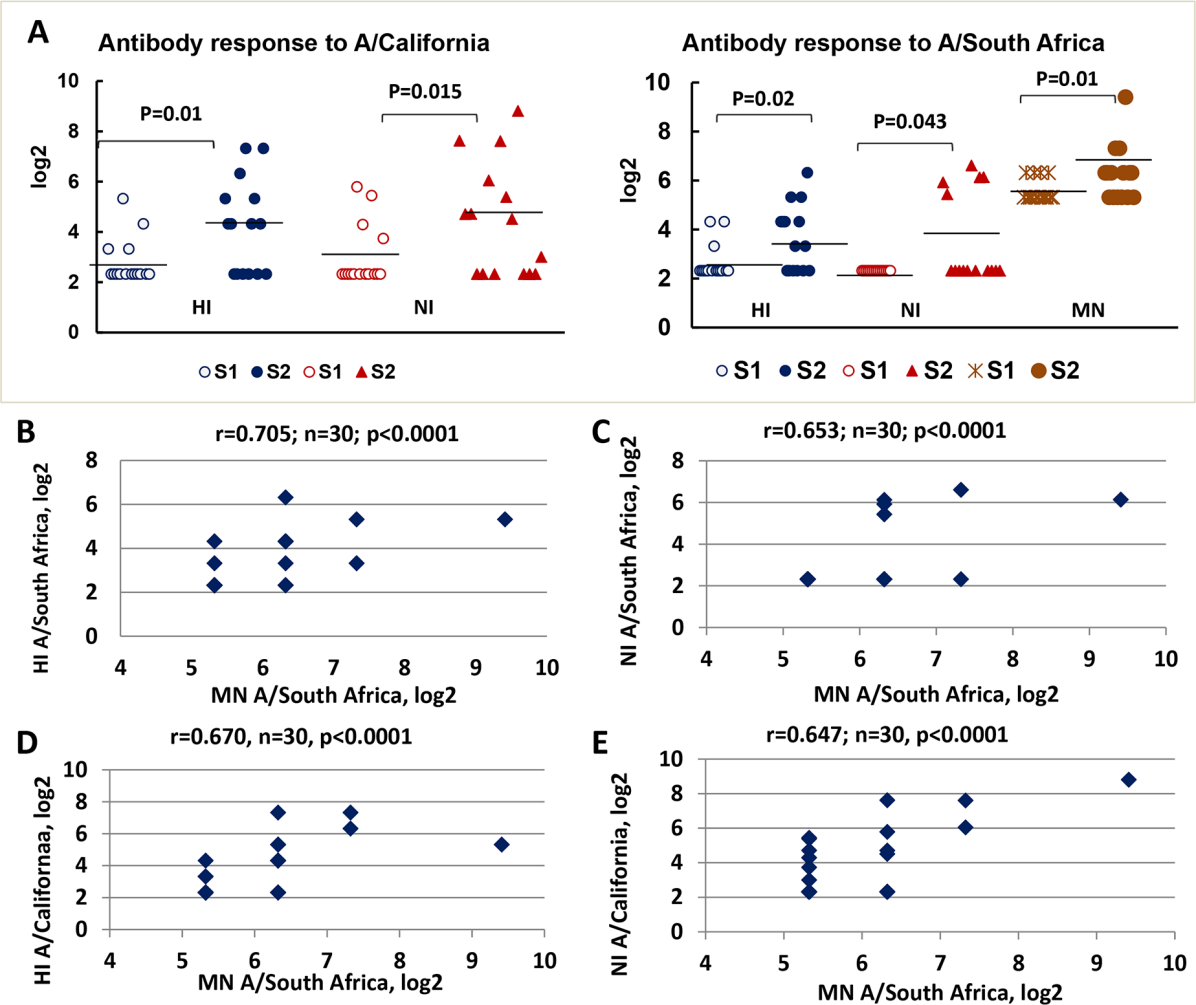
The antibody levels against A/H1N1pdm09 viruses in paired sera from the A/H1N1pdm09-infected patients (n = 15). (A) Increase in antibody titers against A/California/07/2009 (H1N1)pdm as determined by HI and NI assays and against A/South Africa/3626/2013 (H1N1)pdm as determined by HI and, NI, and MN assays. The antibody titers were expressed as log2. (B) Correlation between MN and HI antibodies against A/South Africa/3626/13 (H1N1)pdm in sera collected from the convalescents. (C) Correlation between MN and NI antibodies against A/South Africa/3626/13 (H1N1)pdm in sera collected from the convalescents. (D) Correlation between MN antibodies against A/South Africa/3626/13 (H1N1)pdm and HI antibodies against A/California/07/2009 (H1N1)pdm in sera collected from the convalescents. (E) Correlation between MN antibodies against A/South Africa/3626/13 (H1N1)pdm and NI antibodies against A/California/07/2009 (H1N1)pdm in sera collected from the convalescents. The “r” means Spearman’s rank correlation coefficient.

We included the 15 paired sera from the patients with laboratory-confirmed A/H1N1pdm09 infection. We observed that mean HI and NI antibody titers against A/California/07/09 (H1N1)pdm were significantly higher on days 4–7 after the onset of symptoms compared to those on date of onset (*P* = 0.01, *P* = 0.015, respectively) (Fig 5A). HI and NI antibody titers against A/South Africa/3626/13 (H1N1)pdm increased as the disease progressed (*P* = 0.02, *P* = 0.043). Seven of 15 participants (46%) had the ≥ 4-fold HI antibody increase either against A/California/07/09 (H1N1)pdm or A/South Africa/3626/13 (H1N1)pdm with 6 participants responding simultaneously to both viruses. Sera collected from 5 participants with increased post-infection NI antibodies >1:40 reacted with A/South Africa/3626/13 (H1N1)pdm in the HI test.

Because the virus neutralization by antibodies *in vitro* often reflects the biological effect of protective antibodies and the MN test is known to correlate well with the HI assay [25], we compared results of HI and NI titers with the MN antibody titers determined when A/South Africa/3626/13 (H1N1)pdm was used as an antigen. A strong relationship was found between neutralizing antibody titers and HI antibody titers against A/South Africa/3626/13 (H1N1)pdm (r = 0.705, n = 30, *P* < 0.0001), whereas a medium relationship was found between neutralizing antibody titers and NI antibodies against A/South Africa/3626/13 (H1N1)pdm (r = 0.579, *P* < 0.0001) as determined by Pearson correlation test (Fig 5B and 5C). A medium relationship was observed between neutralizing antibody titers against A/South Africa/3626/13 (H1N1)pdm and HI antibody titers against A/California/07/09 (H1N1)pdm (r = 0.670, n = 30, P < 0.0001) as well as between MN antibody titers against A/South Africa/3626/13 (H1N1)pdm and NI antibody titers against A/California/07/09 (H1N1)pdm (r = 0.647, n = 30, P < 0.0001) (Fig 5D and 5E). These data suggest that anti-NA antibodies may be virus-neutralizing along with anti-hemagglutinating antibodies.

## Discussion

Our data suggested that a small number of patients examined in the present study may have been infected with A/South Africa/3626/13-like influenza viruses by March 2016 and this resulted in the induction of homologous HI and NI antibodies. Contact with previously circulated A/H1N1 viruses did not induce cross-reactive NI antibodies against A/South Africa/3626/13 (H1N1)pdm, but induced NI antibodies cross-reactive with A/California/07/09 (H1N1)pdm. Our survey revealed a low level of detectable NI antibodies against new antigenic A/H1N1pdm09 variant (5.2%), especially in light of the fact that pandemic viruses have been circulating in Russia for more than 6 years. As it was shown in our previous work [26], even in 2005, *i*.*e*. long before the appearance of A/H1N1pdm09 in humans, the level of cross-reactive antibodies against A/California/07/09 (H1N1)pdm NA with titers ≥1:20 was 7.1%, and after the introduction of the virus into circulation it increased up to 30% and later up to 53% in 2016. The levels of “herd” immunity against NA, as determined by the number of subjects with NI antibody titers ≥1:40 against the A/South Africa/3626/13 (H1N1)pdm were low compared to those against A/California/07/09 (H1N1)pdm (Fig 3B). One explanation of these results could be antigenic differences between NAs of the two pandemic viruses. As previously reported, even a single amino acid change within an immunodominant epitope may lead to loss of reactivity with polyclonal antisera [27]. For example, introduction of a single NA amino acid change in A/Solomon Islands/3/2006 strain at position 329 resulted in reduced enzyme inhibition by ferret and human sera directed against this virus [28]. The amino acid sequences of the A/California/07/09 (H1N1)pdm NA and A/South Africa/3626/13 (H1N1)pdm NA differed by 2% and 7 substitutions were located in the head domain, outside the active center. The substitutions N372K and K432E were located in the portion from the second binding site, commonly found in N6 and N9 avian influenza viruses [29]. When MUNANA was used as a substrate, A/California/07/09 (H1N1)pdm NA exhibited significantly decreased NA activity compared to that of A/South Africa/3626/13 (H1N1)pdm (Fig 1C, Table 1). These results may suggest that there is interplay between enzyme activity and antigenicity of N1 NA protein. Our finding may also explain the ineffective inhibition of A/South Africa/3626/13 (H1N1)pdm NA by cross-reactive NI antibodies induced against previously circulated A/H1N1 viruses, including A/California/07/09 (H1N1)pdm.

Previously it has been reported that anti-HA stalk monoclonal antibodies bind to H6 HA reducing the NA activity of the reassortant H6NX viruses through steric interactions [22] thus affecting the results of the NI reaction. Despite the fact that we used A/H6N1 reassortant virus in one case and A/H7N1 in the other case to NI test, the data can still be comparable since the HA stalk domain is highly conservative [30]. We assume that the difference in NI antibody levels against A/California/07/09 (H1N1)pdm and A/South Africa/3626/13 (H1N1)pdm in the population as a whole can be attributed to the presence of cross-reactive, rather than homologous, antibodies to a drift A/H1N1pdm09 variant. This was confirmed by the data from the A/H1N1pdm-positive patients, when the correlation between NI antibody levels against A/California/07/09 (H1N1)pdm and A/South Africa/3626/13 (H1N1)pdm were more pronounced compared to the population at a whole (Fig 3D). Analyzing the sera of patients soon after the influenza infection we showed that the immune responses to both influenza surface antigens were induced simultaneously right after natural infection with A/H1N1pdm09 viruses (Fig 5A and 5B). Some autonomy of the HI and NI immune responses among those subjects who have been exposed to the virus in the past may be related to the timing of the material sampling, particularly when detecting anti-NA antibodies.

Influenza infection represents a particular risk of complications and increased mortality in the elderly. The disease is characterized by a combination of inflammatory changes in the upper respiratory tract with general intoxication and damage to the nervous and cardiovascular systems, thus causing severe complications when developed against the background of atherosclerotic changes in the cardiovascular system and other chronic conditions [31]. In a number of previous studies, the NI antibody titers against A/H1N1pdm09 were detected in the sera of older individuals [32], possibly explaining the reported low incidence of A/H1N1pdm09 disease in the elderly [33]. Indeed, the importance of NA immunity against naturally occurring influenza was demonstrated by evaluating HI and NI antibody titers in a study conducted during 2009–2011 [34] when increased serum NI titers were associated with reduced illnesses. Elderly individuals who were likely exposed to 1918 Spanish Flu pandemic had high neutralizing titers against A/H1N1pdm09 [21] and were more likely to possess cross-reactive antibodies against A/H1N1pdm09 N1 NA. In the present study, we did not observe significant difference in HI and NI antibody levels against A/South Africa/3626/13 (H1N1)pdm among individuals born before 1957 compared to the younger subjects. In contrast, the highest levels of NI antibodies against A/California/07/09 (H1N1)pdm were found in the older age group compared to other groups studied. Thus, the highest levels of NI antibodies against A/California/07/09 (H1N1)pdm may be attributed to cross-reactive antibodies against A/H1N1 viruses other than A/H1N1pdm09. A group of middle-aged patients with the lowest level of NI antibodies against a new antigenic A/H1N1pdm09 variant was identified, indicating the highest-priority vaccination against new antigenic variants.

## Supporting information

S1 Appendix Peroxidase-linked lectin assay to determine neuraminidase inhibiting antibodies using reassortant influenza viruses. dx.doi.org/10.17504/protocols.io.n3adgie.
(PDF)

S2 Appendix HI and NI antibody titers against A/California/07/09 (H1N1)pdm virus in 2009–2010 and 2015–2016 epidemic seasons among participants 20–59 years old. (PDF)

S3 Appendix HI and NI antibody titers against A/South Africa/3626/2013 (H1N1)pdm and A/California07/2009 (H1N1)pdm strains among 134 volunteers examinated in 2016. (PDF)

S4 Appendix The local ethics committee protocol. (PDF)

S5 Appendix The informed consent form. (PDF)

## Abbreviations

ARI: Acute Respiratory Infections
GMT: Geometric Mean Titers
HA: Hemagglutinin
NA: Neuraminidase
HI: Hemagglutination Inhibition
ILI: Influenza-Like Illnesses
NI: Neuraminidase Inhibition
MN: Microneutralization
Me: Medians
