# Supplementary material for: Anti-neuraminidase antibodies against pandemic A/H1N1 influenza viruses in healthy and influenza-infected individuals
Source: PLoS One. 2018 May 9;13(5):e0196771. doi: 10.1371/journal.pone.0196771 (PMC5942809; doi:10.1371/journal.pone.0196771)
Supplement: S1 Appendix — dx.doi.org/10.17504/protocols.io.n3adgie. (PDF) [file pone.0196771.s001.pdf]

Peroxidase-linked lectin assay to determine neuraminidase-inhibiting antibodies using reassortant influenza viruses

Version 2

Yulia A. Desheva,Tatiana A. Smolonogina,Ivan A. Sychev

Abstract

The NA inhibition test using large molecular fetuin substrate is believed to be the most specific method to detect antibodies against influenza virus neuraminidase. To avoid cross-reacting with hemagglutination-inhibition antibodies we have prepared several reassortant A/H7N1 viruses containing the NA of A/California/07/09(H1N1)pdm, A/New Caledonia/20/1999(H1N1) or A/Vietnam/1203/04(H5N1), and the hemagglutinin derived from A/equine/Prague/1/56(H7N7) influenza virus. Adjustment and validation of the NI test with the reassortant viruses included the determination of the viruses working concentration and selection of optimal conditions for the enzymatic reaction.

Citation: Yulia A. Desheva,Tatiana A. Smolonogina,Ivan A. SychevPeroxidase-linked lectin assay to determine neuraminidase-inhibiting antibodies using reassortant influenza viruses. **protocols.io**

dx.doi.org/10.17504/protocols.io.n3adgie

Published: 27 Mar 2018

Before start

Preparation of reagents dilutions

1. Prepare a stock solution of fetuin at 10 mg/ml in coating buffer and store in 500 µl aliquots at -20 °C.
2. Prepare a working solution of fetuin (50 µg/ml) immediately before coating plates by diluting the stock solution 200-fold in coating buffer.
3. Prepare a stock solution of BSA at 100 mg/ml in sterile PBS (sPBS) and store in 10-25 ml aliquots at -20°C.
4. The diluent for virus and sample dilutions (PBS-BSA). Prepare the 1% work solution of BSA (10 mg/ml) right before assay using PBS as a diluent. After thawing aliquot with stock BSA, you may storage (1-2 months) it in a refrigerator at +4°C for next assays.
5. To prepare a stock solution of lectin, mix 1 mg of lectin and 5 ml of sPBS and store at +4°C.
6. Right before use, prepare a work solution of lectin at 2.5 µg/ml by diluting the stock solution 80-fold in PBS-BSA.
7. Prepare a work solution of RDE from original solution by 10-fold dilution at PBS-BSA.
8. Prepare a sufficient volume of wash buffer – 0.5% PBS-Tween 20 (T-PBS). Store at +4°C.
9. Prepare the peroxidase substrate TMB. Place at room temperature for 30 min before use to warm up.
10. Prepare the stop solution - 1 N H_2SO_4 - add 27.2 ml stock 98% H_2SO_4 to 973 ml distilled H_2O.

Serum samples

This is an open access protocol distributed under the terms of the Creative Commons Attribution License, which permits unrestricted use, distribution, and reproduction in any medium, provided the original author and source are credited

1. Heat all sera in a water bath at 56°C for 30 min. Store the sera at -20°C before and after the heat treatment.

Materials

TMB Substrate Reagent Set
555214
Fetuin from fetal bovine serum
F3004
High-binding 96-well microplates
655061
Bovine serum albumin (BSA)
A7030
Peroxidase-conjugated lectin from Arachis hypogaea
L7759
Receptor destroying enzyme (RDE)
View
✓ Sulfuric Acid (H_2SO_4)
✓ 0,1M carbonate-bicarbonate buffer with pH=9.5-9.7
✓ 0,01M phosphate-buffered saline (PBS) with pH=7.3-7.5

Protocol

Step 1.
Adjustment of the virus working dose to use in enzyme-linked lectin assay.

Step 2.

Prepare fetuin-coated plates. Coat 96-well plate with 150 µl of the working solution of fetuin and incubate it at +4°C overnight (at least 18h).

AMOUNT
150 µl Additional info: per well
TEMPERATURE
4 °C Additional info:

NOTES
Yulia Desheva 07 Mar 2018

Still, It is possible to use plates after 2-3 days of incubation.

Step 3.

Prepare viruses dilutions. In 3 short rows of 96-well polymer plates for immunologic reactions with a U-shaped bottom serially two-fold dilutions of the interested virus at PBS-BSA. Titration starts from 130 µl of the virus at concentration of 1024 hemagglutination units (HAU) per 50 µl. Add 65 µl of PBS-BSA to have final volume 130 µl. Use 130 µl PBS-BSA in 4 wells as negative control. Use 130 µl of the work solution of RDE in 4 wells as positive control. Incubate for 30 min. Place the plate in a humidified

This is an open access protocol distributed under the terms of the Creative Commons Attribution License, which permits unrestricted use, distribution, and reproduction in any medium, provided the original author and source are credited

incubator at 37°C. This step imitating contact of the virus with serum.

🌡 TEMPERATURE
37 °C Additional info: in a humidified incubator

Step 4.

Transfer samples with virus to the fetuin-coated plate. While contact is going, wash fetuin-coated plate 2x with sPBS 200 ml/well then invert and pat the plate on absorbent paper towels. After contact, transfer the virus dilutions into the corresponding well rows of a plate with sorbed fetuin using a multichannel pipette of 100 µl starting with the highest concentration. Place the plate in a humidified incubator at 37°C for 1h.

🌡 TEMPERATURE
37 °C Additional info: in a humidified incubato

NOTES
Yulia Desheva 07 Mar 2018

Primarily, It is better to transfer fluid from control wells, then change pipette tips and transfer the virus starting from highest dilution.

Step 5.
Add the lectin from Arachis hypogaea. When the incubation is complete discard liquid from wells. Wash 4x with T-PBS 200 ml/well. Add to each well 100 µl lectin. Place the plate at room temperature for 1h.

AMOUNT
100 µl Additional info: per well

🌡 TEMPERATURE
20 °C Additional info: at RT

Step 6.
Complete the assay. When the incubation is complete discard liquid from wells. Wash 4x with T-PBS 200 ml/well. Add to each well 100 µl TMB. Incubate for 2-3 min (maybe more or less) at room temperature. Stop the reaction by adding to each well 100 µl 1 N H_2SO_4. Measure the optical density (OD) at 450 nm using the universal plate reader.

Step 7.

Data analysis. Define the yield of the reaction product (RY) as follows: RY = (OD-VD – OD-NC)/(OD-PC – OD-NC)*100%, where OD-VD – is a mean of OD from wells with virus dilution; OD-NC – is a mean of OD from wells with negative control; OD-PC – is a mean of OD from wells with positive control. Determination of the range of linear dependence of the yield of the sialidase reaction product on a concentration of a virus.

NOTES
Yulia Desheva 07 Mar 2018

Usually this range of linear dependence lasts from 8 HAU/50µl to 128 HAU/50µl. 128 HAU/50µl is the upper limit of a working concentration of a virus.

Step 8.

This is an open access protocol distributed under the terms of the Creative Commons Attribution License, which permits unrestricted use, distribution, and reproduction in any medium, provided the original author and source are credited

Enzyme-linked lectin assay.

Step 9.

Prepare fetuin-coated plates. See step 2.

Step 10.

Prepare sample dilutions. In short rows of 96-well polymer plates for immunologic reactions with a U-shaped bottom in 65 µl, serial two-fold dilutions (from 1/10 to 1/640) of the serum samples to be tested were made at PBS-BSA. In each plate, the same positive and negative reference sera are titrated. NB! Don’t forget to vortex all sera samples before adding it in plates. Prepare a dilution of a virus at 128 HAU in 50 µl by PBS-BSA. Add 65 µl of the diluted virus into each well. The last row of 96-well plates (H-row) is used for positive and negative virus control (65 µl of the diluted virus + 65 µl PBS-BSA and 130 µl PBS-BSA respectively). Incubate for 30 min. Place the plate in a humidified incubator at 37 °C.

🌡 TEMPERATURE

37 °C Additional info: in a humidified incubato

Step 11.

Complete the assay by following steps 4 – 6.

Step 12.

Data analysis. The result of the enzymatic reaction estimated as OD data is determined for a series of dilutions of each blood serum. Use the OD set to plot the residual NA enzymatic activity in the presence of anti-NA antibodies, calculated according to the formula below: Activity = (ODsample – OD-NC)/(OD-PC – OD-NC)*100%, where: OD-PC and OD-NC are a mean of OD from wells with positive control and negative control. Determine the titer of serum anti-NA antibodies as the reciprocal dilution of the sample causing 50% inhibition of NA activity. To calculate the 50% inhibitory concentration (IC50) use regression analysis. Rough nonlinear regression curve fitting can be performed by Microsoft-Excel. Precise curve fitting can be performed by Wolfram-Alpha web-tool.

NOTES

Yulia Desheva 08 Feb 2018

If IC_{50} was not achieved at any dilution, the titer will be less than the first dilution. If the first dilution is 1:10, then the titer will be <1:10.

Step 13.

Warnings

All live reassortant viruses must be handled using biosafety level 2 (BSL2)-enhanced practices in a laboratory.

This is an open access protocol distributed under the terms of the Creative Commons Attribution License, which permits unrestricted use, distribution, and reproduction in any medium, provided the original author and source are credited
