# Supplementary material for: Anti-neuraminidase antibodies against pandemic A/H1N1 influenza viruses in healthy and influenza-infected individuals
Source: PLoS One. 2018 May 9;13(5):e0196771. doi: 10.1371/journal.pone.0196771 (PMC5942809; doi:10.1371/journal.pone.0196771)
Supplement: S2 Appendix — (PDF) [file pone.0196771.s002.pdf]

#	Age	Reciprocal antibody titers against A/California/07/09 in 2009-10		#		Reciprocal antibody titers against A/California/07/09 in 2015-16	
		HI	NI		Age	HI	NI
1	20	5	11	1	24	20	5
2	20	40	73	2	24	80	118
3	20	20	24	3	26	10	11
4	20	10	43	4	29	10	21
5	22	5	5	5	31	10	37
6	22	40	24	6	31	40	5
7	24	320	74	7	32	40	9
8	24	40	32	8	33	40	29
9	26	40	44	9	33	5	70
10	26	40	55	10	34	5	5
11	26	10	5	11	34	5	66
12	29	40	26	12	36	20	9
13	29	160	46	13	38	5	11
14	35	5	5	14	40	5	145
15	39	320	134	15	40	5	29
16	40	5	5	16	40	5	17
17	40	5	5	17	41	20	22
18	41	40	5	18	41	20	5
19	42	5	5	19	42	10	10
20	42	5	8	20	43	10	5
21	43	40	5	21	43	20	5
22	44	5	9	22	43	5	5
23	44	80	12	23	43	20	11
24	45	5	17	24	45	5	9
25	45	5	5	25	46	10	44
26	45	40	5	26	48	10	5
27	46	5	5	27	48	5	5
28	46	160	56	28	51	40	5
29	48	80	6	29	51	5	5
30	49	5	11	30	51	5	47
31	49	5	5	31	52	20	37
32	50	5	5	32	53	10	9
33	50	10	5	33	54	5	5
34	51	5	5	34	54	5	5
35	52	5	5	35	54	5	14
36	52	5	5	36	56	5	58
37	52	10	32	37	56	10	5
38	54	5	5	38	57	5	5
39	55	5	19	39	57	10	5
40	56	10	180	40	57	5	5
41	58	10	72	41	57	10	13
42	58	5	5	42	58	20	5
				43	58	10	5
				44	59	10	62

				45	59	10	29
				46	59	20	5
				47	59	10	5
