# Supplementary material for: Anti-neuraminidase antibodies against pandemic A/H1N1 influenza viruses in healthy and influenza-infected individuals
Source: PLoS One. 2018 May 9;13(5):e0196771. doi: 10.1371/journal.pone.0196771 (PMC5942809; doi:10.1371/journal.pone.0196771)
Supplement: S3 Appendix — (PDF) [file pone.0196771.s003.pdf]

#	HI antibodies		NI antibodies	
	A/South Africa/3626/13	A/California/07/09	A/South Africa/3626/13	A/California/07/09
1	10	5	5	85
2	10	5	5	14
3	5	5	5	82
4	20	20	5	9
5	5	5	5	147
6	5	5	5	5
7	5	5	5	8
8	40	40	5	34
9	5	5	5	33
10	10	20	5	53
11	5	5	5	5
12	5	20	5	27
13	5	5	5	19
14	10	5	5	66
15	10	5	129	545
16	10	10	5	5
17	5	5	11	106
18	5	5	5	58
19	5	5	5	23
20	10	10	5	62
21	10	5	5	5
22	20	5	5	17
23	5	40	5	5
24	20	5	5	66
25	10	10	5	37
26	40	5	20	331
27	5	10	5	5
28	5	10	5	25
29	5	5	53	556
30	5	5	5	27
31	40	40	5	5
32	10	20	5	26
33	5	5	5	38
34	10	5	5	122
35	5	10	5	320
36	5	10	5	13
37	5	10	5	5
38	5	5	5	76
39	5	5	5	21
40	5	5	5	47
41	5	5	5	110
42	5	5	5	5
43	5	5	5	5
44	10	20	5	37

45	20	10	5	29
46	5	5	5	27
47	10	5	5	5
48	10	10	5	38
49	5	10	5	53
50	20	20	5	26
51	5	5	5	10
52	5	5	5	106
53	10	10	5	77
54	10	10	5	5
55	5	5	5	5
56	5	5	5	5
57	80	160	10	718
58	5	10	5	19
59	5	10	5	160
60	5	10	5	44
61	10	20	5	17
62	5	5	5	9
63	5	10	5	5
64	5	5	5	5
65	5	10	14	258
66	5	20	5	122
67	5	5	5	30
68	10	10	5	26
69	5	5	5	72
70	5	5	5	44
71	40	5	5	145
72	10	5	5	47
73	5	5	5	5
74	5	5	5	55
75	5	5	5	5
76	5	10	5	5
77	10	10	5	102
78	5	5	5	30
79	5	5	5	47
80	5	10	5	5
81	5	5	5	17
82	5	5	5	5
83	5	5	5	12
84	5	5	5	5
85	5	10	5	13
86	5	5	5	5
87	10	20	5	55
88	5	10	5	5
89	5	5	5	5
90	5	5	5	5
91	5	5	26	253

92	5	20	5	103
93	10	5	5	5
94	5	5	5	11
95	10	20	5	11
96	5	10	5	9
97	5	20	5	5
98	10	40	5	5
99	5	5	5	31
100	20	40	16	160
101	10	20	5	27
102	20	40	5	56
103	20	20	40	623
104	5	10	5	5
105	5	10	5	21
106	5	5	5	14
107	5	5	5	5
108	5	5	5	10
109	10	20	5	22
110	10	20	5	5
111	10	20	5	52
112	5	5	5	5
113	5	5	5	11
114	5	5	9	59
115	5	40	5	29
116	5	20	5	5
117	5	5	27	70
118	10	40	12	9
119	5	20	5	5
120	5	10	5	11
121	5	5	5	29
122	40	80	5	118
123	40	80	37	640
124	20	40	5	129
125	5	5	5	103
126	5	5	5	17
127	10	20	5	5
128	5	10	5	10
129	5	10	5	5
130	5	40	5	36
131	5	10	5	5
132	5	10	5	5
133	10	20	5	11
134	5	20	5	35
