# Supplementary material for: Anti-neuraminidase antibodies against pandemic A/H1N1 influenza viruses in healthy and influenza-infected individuals
Source: PLoS One. 2018 May 9;13(5):e0196771. doi: 10.1371/journal.pone.0196771 (PMC5942809; doi:10.1371/journal.pone.0196771)
Supplement: S4 Appendix — (PDF) [file pone.0196771.s004.pdf]

Federal State Budgetary Scientific Institution «Institute of Experimental Medicine»

(FSBSI «IEM»)

Local Ethics committee

**Extract from the protocol № 2/16**

12.05.2016 14:00

Address: 71, Kamennoostrovsky Avenue, St. Petersburg, Russia, 197022

Phone: 8 (812) 234-29-00

**The following members attended:**

The chairman – A.V. Shabrov

The secretary – D.E. Korzhevskii

M.D. Didur, A.V. Suvorov, V.M. Klimenko, A.D. Denisenko, V.B. Vasiliev, P.G. Nazarov

**Listened at a meeting:** materials of the initiative research project "Study of the formation of antibodies to neuraminidase of influenza virus in case of infection and immunization with influenza vaccines"

Supervisor – Y.A. Desheva

**Reviewed documents:**

-description of the research

**Decision:**

To approve an initiative research project "Study of the formation of antibodies to neuraminidase of influenza virus in case of infection and immunization with influenza vaccines" (Supervisor – Y.A. Desheva).

Vote: "yes" – unanimously

The chairman of the Local Ethics committee

of the FSBSI «IEM»

/A.V. Shabrov/

The secretary of the Local Ethics committee

of the FSBSI «IEM»

/D.E. Korzhevskii/
