# Supplementary material for: Anti-neuraminidase antibodies against pandemic A/H1N1 influenza viruses in healthy and influenza-infected individuals
Source: PLoS One. 2018 May 9;13(5):e0196771. doi: 10.1371/journal.pone.0196771 (PMC5942809; doi:10.1371/journal.pone.0196771)
Supplement: S5 Appendix — (PDF) [file pone.0196771.s005.pdf]

Medical scientific Center
Federal State budget scientific institution
Institute of Experimental Medicine

**Informing voluntary consent to medical intervention**

I, ______________________________________________________________________________
(Full name)
_____ year of birth, residing at _________ St. Petersburg ________________

______________________________________________________________________________

This section is to be completed by the legal representative of the patient
The legal representative of the patient
Parent (mother or father) if the patient is a minor under the age of 15;
The adopter legally;
A guardian / trustee, who is authorized on the basis of an administrative act (if a citizen is legally recognized)
Ineffective or limited in capacity)
The Attorney

_________________________________________________________ / ____________
(Signature)
Passport series ______ No. _____________ Issued by

________________________________________
Date of issue of "____" _________________ 20 ____ g. Registered at ___________________
____________________________________ Place of
work__________________________________
Phone house (____) ________________; mob (____) ____________ E mail
______________________

The attending physician _______________ / ___________________________ / "____" ___________
20 ___ g.
Signature (Full name)

- According to my will, full and comprehensive explanations are given of the nature, severity and possible complications of my disease (health of the patient) established in the given medical and preventive institution, and I undertake to observe;
-I am acquainted with the schedule and rules of the treatment and protection regime,
- I kindly give my consent to carry out diagnostic tests for me (represented), in accordance with the doctor's prescriptions, for general and biochemical blood tests, blood tests for the presence of human immunodeficiency virus, viral hepatitis, pale treponema, general urine analysis, electrocardiography, X-ray, ultrasound and endoscopic research and treatment - taking tablets, injections, intravenous injections, diagnostic and therapeutic punctures, physiotherapy their procedures. The need for other methods of examination and treatment will be explained to me in addition.
-I am informed of the goals, nature and adverse effects of diagnostic and treatment procedures, the possibility of unintentional injury to health, as well as about what is to be done by me (represented) during the conduct.
-I am informed (informed) that it is necessary for me (a representative) to take prescribed medications and other methods of treatment on a regular basis, immediately inform the doctor of any deterioration of health, agree with the doctor to take any medications that are not prescribed.
-I am warned (warned) and I realize that refusal of treatment, non-observance of the curative-protective regime, recommendations of medical workers, the mode of taking medications, unauthorized use of medical instruments and equipment. Uncontrolled self-medication can complicate the process of treatment and adversely affect the health status,
-I put (informed) the doctor about all the health problems, including allergic manifestations or individual intolerance to medications, about all the traumas, operations, diseases, environmental and production factors of physical , chemical or biological nature, affecting me (represented) during life, about the drugs being taken. I informed (reported) truthful information about heredity, as well as about the use of alcohol, narcotic and toxic drugs.
- I ________ (agree) to inspection by other medical workers and students of medical schools and colleges exclusively for medical, scientific or educational purposes, taking into account the preservation of medical secrecy.

-I am acquainted and agree with all the points of this document, the provisions of which are explained to me, I understand them, and voluntarily give my consent for examination and treatment in the proposed volume,
-I will allow, if necessary, to provide information about my diagnosis, severity and nature of my illness to my relatives, legal representatives,
Citizens

______________________________________________________________

______________________________________________________________

______________

x
"___" _________________ 20 ______ year Signature of patient's legal representative

Signed in my presence
x
Doctor ________________________________________ (signature)
(Position, Name)
Note
The consent for medical intervention (treatment) for persons under the age of 15 years and citizens recognized as legally incapacitated by law are given by their legal representatives (parents), adopters, guardians or custodians) indicating the name, passport data, relationship after the communication they are informed about the results of the examination, the presence of the disease, its diagnosis and prognosis, the methods of treatment, the risk associated with them, possible options for medical intervention, their consequences, and the results of the treatment.
In the absence of legal representatives, a decision on the need for treatment takes a consultation, and if it is not possible to collect a consultation, the directly attending physician with the subsequent notification to the head of the MSC "IEM".
In cases where the condition of a citizen does not allow him to express his will, the need for treatment is urgent, the question of medical intervention in the interests of the citizen is decided by the council, and if it is not possible to collect the consultation, the directly attending physician with the subsequent notification to the head of the MSC "IEM".
Additional Information

______________________________________________________________

______________________________________________________________

______________________________

______________________________________________________________

________

______________________________________________________________

________

______________________________________________________________

________

______________________

x
"___" _________________ 20 ___ years Signature of patient's legal representative
Signed in my presence a doctor

______________________________________________________

x

(Position, full name) signature

федеральное государственное бюджетное научное учреждение «Институт экспериментальной медицины» (ФГБНУ «ИЭМ») ОГРН 1037828000198
ДЛЯ ДОКУМЕНТОВ
